# Investigating adult male sexual function and fertility after childhood hypospadias repair surgery – a systematic review

**DOI:** 10.1186/s12894-026-02104-6

**Published:** 2026-03-23

**Authors:** Safendra Siregar, Ricky Adriansjah, Steven Steven, Kenfin Surya

**Affiliations:** 1 Department of Urology, Hasan Sadikin Academic Medical Center, Universitas Padjadjaran, Bandung, Indonesia; 2 Department of Urology, Universitas Padjadjaran Academic Medical Center, Sumedang, Indonesia

**Keywords:** Hypospadias, Fertility, Sexual function, Male

## Abstract

**Introduction:**

Hypospadias, a congenital condition where the urethral opening is on the ventral of the penis, presents challenges in both diagnosis and treatment. Despite surgical advancements, long-term issues such as psychological distress and concerns about sexual function and fertility persist. Our study aims to systematically review sexual function and fertility outcomes in adults who underwent childhood hypospadias repair surgery, offering insights for clinical practice and future research.

**Materials and methods:**

This is a systematic review study. Following Preferred Reporting Items for Systematic Reviews and Meta-Analysis guidelines, we searched PubMed, Embase, and Scopus using predefined relevant keywords. Quality assessment was conducted using the Newcastle-Ottawa Scale.

**Results:**

Among 288 initially identified articles, 14 meet eligibility criteria with a total of 6,737 participants for fertility outcomes and 933 for sexual function outcomes. Overall included study quality was deemed average. Distal hypospadias showed more favorable sexual function and fertility compared to severe cases that may experience issues such as ejaculation problems and infertility.

**Conclusion:**

Our review suggests that individuals who underwent childhood urethral repair surgery due to distal type hypospadias have more favorable sexual and reproductive outcomes than proximal hypospadias. However, proximal cases may exhibit suboptimal sexual function due to penile curvature, lower semen quality, and ejaculation function. Further multicentric research is needed to confirm these findings and guide clinical management effectively.

**Supplementary Information:**

The online version contains supplementary material available at 10.1186/s12894-026-02104-6.

## Introduction

Hypospadias is a congenital malformation, in which the meatus is opening on the ventral aspect of the penis [1]. The meatus may be positioned at various levels. Hypospadias is the second most frequent congenital abnormality in males after cryptorchidism. The abnormality is typically more complicated and may be accompanied by abnormalities of the skin, a narrow or large meatus, penile curvature, or glans distortion. Other chromosomal or urogenital defects may appear in severe situations [2]. This aberration is thought to have a complex etiology that includes genetic, endocrine, and environmental components [3].

The prognosis of hypospadias has significantly improved over the past three decades, but for long term outcome, many adults still have some sort of psychological issue with the cosmesis, and others still have unsatisfactory sex lives [4]. Most research nowadays focused on urinary function outcome (voiding) but left the sexual function in hypospadias follow-up. This is mostly due to the effort needed to evaluate the long-term effect of hypospadias repair would require long term follow-up as well as patient-oriented problems such as low response rates or unwillingness to cooperate in long term follow-up. Therefore, no surprisingly that literature concerning on hypospadias sexuality is scarce [5]. One of the puzzling issues usually brought up by patients after hypospadias repair is sexual function and fertility aspect. The child’s future sexual function and fertility becomes a significant concern as they mature into adults [6]. Therefore, we planned to perform systematically review on sexual function and fertility in adult with history of childhood hypospadias repair surgery.

## Materials and methods

This systematic review study was conducted based on Preferred Reporting items for Systematic Review and Meta-Analysis (PRISMA) 2020 guidelines with a systematic search and the protocol was registered in PROSPERO (CRD420251247553) [7, 8].

### Search strategy

Following PRISMA guidelines, we conduct a literature search on Scopus, Embase, and PubMed, to identify relevant articles which have been published up to the present year. We performed an initial literature search in January 2024 and updated it in November 2025. The search was conducted in English, with terms including (“sexual function” OR “sexual life” OR “erection” OR “ejaculation” OR “fertility” AND “hypospadias” AND “adult”). The search was performed with a combination of some or all these keywords, both in the title and abstract of the article. Search is limited to publications up to November 2025. We followed the Cochrane Handbook for systematic reviews of interventions guidelines for the search [9].

### Eligible criteria

Study designs included in this study were before-and-after studies with or without controls, retrospective and prospective cohort studies, interrupted time series analysis, and randomized controlled trials written in English. Studies were included if there were sexually active adults (age 18–35) with history of childhood (before puberty) hypospadias repair. However, studies with young adults age group (16–18) or older than 35 years old can be included when relevant sexual function data were extractable which will be critically discussed among authors. Literature review articles, case series, letters, notes, conference abstracts, and conference articles were excluded.

### Study selection and extraction

After removing duplicate study, all titles and abstract screening and data extraction were conducted independently by one reviewer. All excluded records were subsequently checked and verified by a second reviewer independently. Any disagreements were resolved through discussion, and when consensus could not be reached, a third senior reviewer adjudicated the final decision. The full text of the studies was evaluated to determine if they were eligible to be included in the review or not. Data were extracted using a standardized table that includes the name of the authors, year of publication, study setting, number of subjects, and the key findings of each study. The results obtained in the included studies will be compared with those of other systematic reviews and literature.

### Quality assessment and risk of bias

Cohort study, both prospective and retrospective, along with case-control study were evaluated using the Newcastle-Ottawa Scale (NOS) for their quality. The studies were judged based on three standpoints which are study group selection, comparability between groups, and the assessment of exposure and outcome of interest. For risk of Bias evaluation categorized into low risk (score ≥ 7), moderate risk (score 4–6) and high risk (score ≤ 3) [10].

## Result

### Study selection

**Fig. 1:**
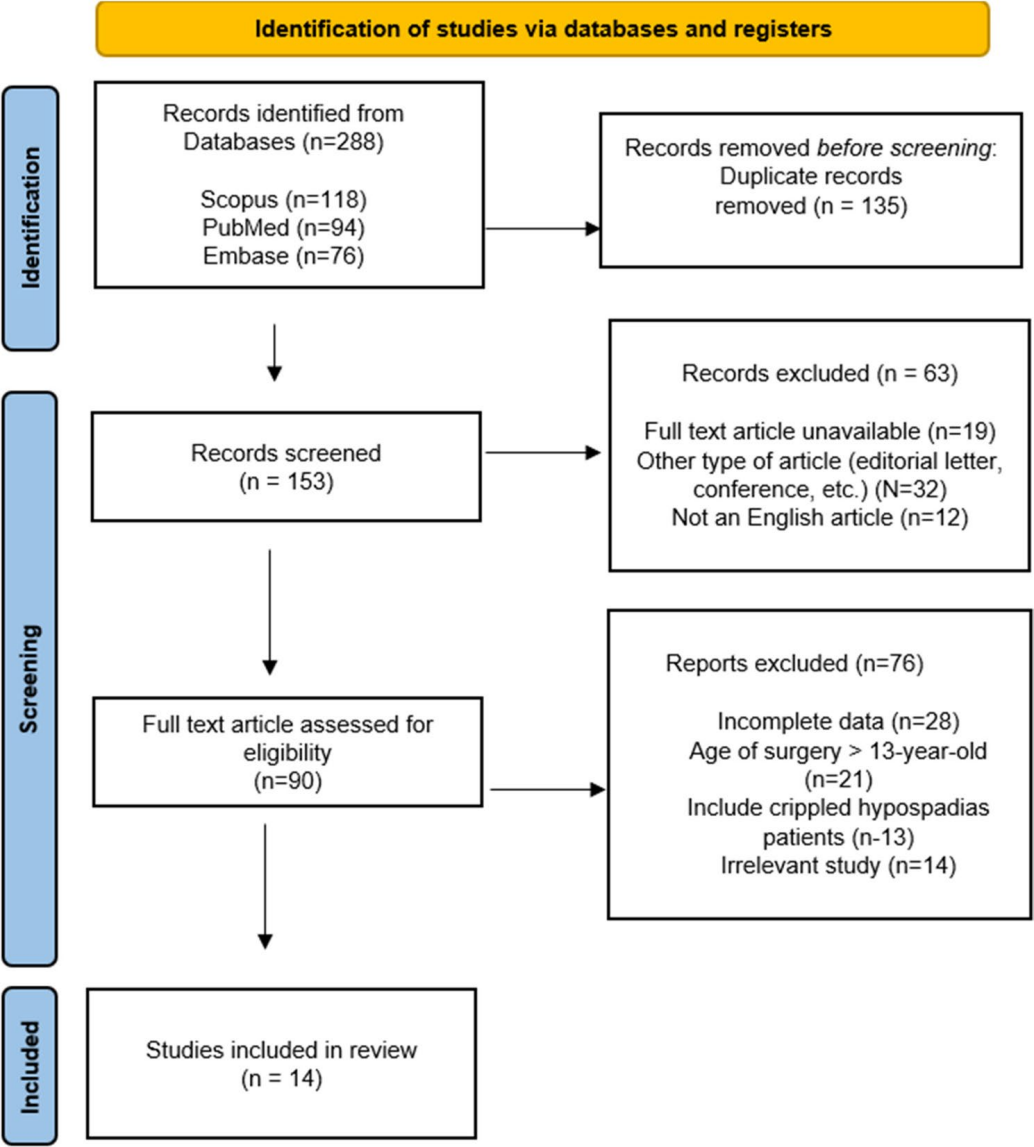
Study selection using Preferred Reporting items for Systematic Review and Meta-Analysis flow diagram

A systematic search was carried out and yielded 288 articles. A total of 153 articles remained, after rechecking and excluding duplicated articles. Sixty-three articles were removed during screening due to inaccessible full text version, not English article, and other type of article. A total of 90 articles were eligible for full-text review. Then, after a comprehensive review of the full-text articles, 76 articles were excluded due to several reasons, and the remaining 14 articles were included in this study (Fig. 1).

### Study characteristics

The study characteristics and summary are shown in Tables 1 and 2, and 3. Of fourteen included studies, we found 10 studies analyzed the sexual function aspect, 3 studies did the fertility, and 1 study did both. The number of included participants in each study varied with total of 6737 males in fertility study and 933 patients for sexual function study. Patients in the study were sexually active age.

**Table 1:** Sexual function Study Characteristics and Finding

Author, Year	Sample characteristic	Hypospadias Classification	Time of Surgery (children / adulthood)	Type of Surgical Approach	Sexual Dysfunction outcome
Tack, 2020	193 patients and 50 control. Mean age: 18.14 years old Control 19.57 years old	132 Distal 38 Midshaft 23 Proximal	Childhood Hypospadias Repair Median: 1.5years (0.1–12)	Both (one stage and two-staged) repair based on urethral defect	• 90% of cases are satisficed sexual function. • Mild Erectile and ejaculation problems occurred in 10% cases
Diaw, 2020	3 of 55 patients Median age: 6 years	Distal Midshaft Proximal *Number not stated	Childhood Hypospadias Repair	Single step repair	• Out of three patients followed up for sexual function, one patient had full score of IIEF-5 (score 12) and average in the other two cases (score 9 each)
Tack, 2021	193 patients and 50 control. Age: Case 18.1 years old Control 19.6 years old	132 Distal 38 Midshaft 23 Proximal	Childhood Hypospadias Repair, performed at age (median 1.5 years old	Primary hypospadias repair and consist of • One intervention • Multiple reinterventions	• Disturbed urinary and/or suboptimal sexual functional outcome was seen in 52.9% of cases. • good sexual outcome was defined as having a straight erection ◊ achieved in 47,1% • 99 of 187 cases (52.9%) had a suboptimal combined urinary and/or sexual functional outcome, opposed to 10 of 49 controls (20.4%).
Husmann, 2020	100 patients Mean age: 30.1 years old	24 Distal 11 Mid-shaft 17 Proximal 48 Penoscrotal	Childhood Hypospadias Repair	Multiple staged hypospadias repair with median number of surgery 5 times	• 37% (37/100) of patients complained of moderate to severe Erectile Dysfunction (SHIM score 16) • The early onset of ED in patients that failed multiple attempts at hypospadias repair in childhood is associated with advancing age, division of the urethral plate, and prior ventral corporal grafting.
Rynja, 2009	66 patients vs. 151 controls Mean age of study 22 years old	47 Distal 8 Midshaft 11 Proximal	Childhood hypospadias repair Median age: 2.1 years old	47% had a single staged operation procedure. Technique of surgery 48% use Mathieu like and 16% prepuce assisted	• Mean IIEF score 20.6 vs. 22.4. • 40% dysfunction in case group vs. 38% in control group
Kilic, 2023	30 patients Mean age ± 27 years old	Distal hypospadias Proximal hypospadias *Number not stated	Childhood Hypospadias Repair	One staged for distal hypospadias Two-staged for proximal hypospadias	• Sexual function in distal hypospadias: similar to normal healthy individuals • In proximal hypospadias : 75% of the patients have symptomatic erectile dysfunction and a higher rate of ejaculation problems
Rynja, 2018	54 patients and 148 controls Median age 18.3 years old	12 Distal hypospadias 42 distal and midshaft hypospadias	Childhood hypospadias repair with median age 1.3 years old	TPIT for proximal hypospadias	• No significant IIEF differences • Noticeable impaired orgasmic function in proximal group
Andersson, 2016	64 patients and 25 controls Median age for proximal hypospadias 17.5 years old compared to 19 years old distal hypospadias and 18 years old control.	33 Proximal 31 Distal	Childhood hypospadias Age not stated	TIP Duckett	• 10% Occasional erectile difficulty in the proximal group
Majstorovic, 2020	63 patients and 60 controls Mean age 21 years old	41 distal 10 midshaft 9 penoscrotal 3 scrotal	Childhood hypospadias with mean age 20.76-month-old (1.7 years old)	TIP with or without dartos flap Skin flap urethroplasty Buccal mucosal graft and skin flap urethroplasty	• Significant difference in frequency of sexual activity and orgasmic ability • No significant in sexual desire, arousal and erectile abilities
Örtqvist, 2017	167 patients and 169 controls Mean age 34 years old	63% distal hypospadias 24% midshaft 13% proximal hypospadias	Childhood surgery means age 5 years old	n/a	• Problems in achieving erection were rare. • Anejaculation found in 6 hypospadias patients. • Glanular sensitivity reported lower in hypospadias patients
Anttila, 2025	196 patients and 1935 controls Median age 16.2 years	96 distal 20 midshaft 53 proximal	Childhood surgery with median age 1.4 years	62 patients had Nesbid-like plication	• All patients reported erections. • 73% achieved EHS 4 (completely hard and fully rigid). • 95% reported a straight penis in erection. • 95% reported successful ejaculation. • Only 3% reported any penile pain (none during erection). • Hypospadias severity and curvature correction did not worsen sexual outcomes.

**Table 2:** Sexual function: Erection and ejaculatory function evaluation

Author, Year	Erectile function	Ejaculation	Others component
Tack, 2020	10 out of 89 (11.2%) have IIEF-5 score 19–21 (mild sexual dysfunction) Severity of hypospadias did not affect IIEF-5 score. Number of penile surgeries, age of first surgery and SPL have no correlation to erectile function	6 occasional anejaculation 3 ejaculation precox / tarda 1 loss of erection 1 split ejaculation from fistula 2 pain 1 sensory problem 12.3% post orgasmic milking	
Diaw, 2020	Only 3 accepted the sexual function survey (delay after surgery was between 2 to 10 years) Best at IIEF-5 score 12. Average 2 others were 9.	n/a	
Tack, 2021	11.2% cases of mild ED	23 cases ejaculatory problem	23.3% had sexual function problems in the case group compared to 6% at control. Suboptimal sexual function (chordee > 30^o^, erectile and ejaculatory problem) more common in proximal vs. distal hypospadias (39.1% vs. 16.8%) 99 of 187 cases (52.9%) had combined urinary and sexual function problem.
Husmann, 2020	37 of 100 patients had erectile dysfunction with 35% moderate ED and 65% severe ED based on SHIM score. 62% responded to oral pharmacologic agent either testosterone plus PDE-5 inhibitors, or PDE-5 inhibitors alone.	n/a	
Rynja, 2009	The IIEF score was 20.6 ± 10.53 and 22.5 ± 9.38 for hypospadias and control. No difference in erectile function between patients and control No significant difference between severity of hypospadias and erectile function after surgery.	Patients especially with proximal hypospadias reported worse ejaculatory function than control	Case group also report higher orgasmic problem than control, maybe due to insecure feeling or shame with penile appearance
Kilic 2023	Erectile function in the distal hypospadias group is similar to normal healthy individuals. In the proximal hypospadias group, approximately 75% of the patients have symptomatic erectile dysfunction.	Ejaculation functions in the distal hypospadias group are similar to normal healthy individuals. In the proximal hypospadias group, approximately 75% of the patients have a higher rate of ejaculation problems.	More than two-thirds of the patients who underwent surgery due to hypospadias in childhood are not very satisfied with their cosmetic penis shape when they reach adulthood. However, the satisfaction rate is much lower in those operating for proximal hypospadias.
Rynja, 2018	No difference between proximal, distal, and control group	4/9 (44.4%) patient had dripping ejaculation in proximal groups compared to 4/38 (10.5%) in distal group	Lower orgasmic function found in proximal hypospadias group with lower sexual desire. Sexual milestones were reach at older age in proximal group compared to distal groups.
Andersson 20,216	Occasion erectile difficulty reported in 10% proximal hypospadias group and 4% in control group	Anejaculation was reported in 11% proximal group and 3% in control.	Both groups were equally satisfied with axis during erection although curvature found in 50% of proximal patient. No difference in perceived glanular sensation, satisfaction with glanular sensation, and ability to have orgasm
Majstrovic 2020	No difference between group There was strong positive correlation with orgasmic ability	N/a	Frequency of sexual activities, arousal and orgasmic ability were significantly complained in patient group. Surgically treated proximal (scrotal) hypospadias had highest values for all domains in GSF scores compared to distal, midshaft, and penoscrotal.
Örtqvist , 2017	No significant difference in erection between groups Impotence reported in 8 patients and 13 control	6 patients complained of anejaculations	Glanular sensitivity was reported lower in hypospadias patient (*p* = 0.001) Sensitivity decreased with the severity of hypospadias. 8 patients report pain during intercourse
Anttila 2025	Strong evidence that postoperative erectile rigidity and penile straightness in adolescence are generally excellent, even in proximal cases.	N/a	

**Table 3:** Fertility and Paternity Study Characteristics and Finding

Author, Year	Sample characteristic	Hypospadias Classification	Time of Surgery (children / adulthood)	Paternity	Fertility aspect
Akslund 2009	112 patients and 318 controls Median age 28.4	54.5% Glanular 28.6% Penile 2.7% Scrotal 14.3% Unclassified	Childhood Age not stated	Paternity 24% vs. 29.4% (*p* < 0.01) ART 13% isolated hypospadias, 43% with associated disorder	• Semen volume (mL) mean 3.5 vs. 3.4 • Sperm concentration (mill/mL) 62.9 vs. 56.0 • Total sperm count (mill) 211 vs. 181 • Motile sperm (%) 63 vs. 69 • Normal morfology (%) 8.4 vs. 7.9
Kumar 2016	73 patients and 70 controls Mean age 23.73 years old	15% Coronal 46% Distal shaft 19% Midshaft 10% Proximal shaft 10% Penoscrotal	30/73 childhood surgery 43/73 adulthood surgery Age not stated	n/a	• Mean semen concentration (mill/mL) 58.88 vs. 68.67 (*p* = 0.02) • Mean semen volume (mL) 1.58 vs. 1.7 (*p* = 0.192)
Örtqvist, 2017	167 patients and 169 controls Mean age 34 years old	63% distal hypospadias 24% midshaft 13% proximal hypospadias	Childhood surgery mean age 5 years old	Paternity 45% vs. 39% (*p* = 0.001) ART: stated as rare	n/a
Nordenvall 2020	6388 patients and 1,275,423 controls Mean age 26.6 years old	57.7% Distal hypospadias 6.0% Proximal hypospadias 36.3% not specify	n/a	Paternity 21.2% vs. 27.1% ART 1.6% vs. 1.6%	Diagnosed as infertility 1.8% vs. 1.6%

## Quality Assessment

We performed evaluation of quality and risk of bias using NOS. [10]. A summary of NOS scores is presented in Table 4. The quality was considered average for the included studies as 7 studies considered low risk for bias and 7 studies had moderate risk. Therefore, the result showed a relatively moderate risk of bias especially in sexual function section.

**Table 4:** The risk of bias assessment using the Newcastle-Ottawa scale

Study	Selection	Comparability	Outcome/Exposure	Score
Tack, 2021	**ΡΡΡ**	**Ρ**	**ΡΡ**	**6**
Diaw, 2020	**ΡΡ**		**ΡΡ**	**4**
Tack, 2020	**ΡΡΡ**	**Ρ**	**ΡΡΡ**	**7**
Husmann, 2020	**ΡΡ**		**ΡΡ**	**4**
Rynja, 2009	**ΡΡΡ**	**Ρ**	**ΡΡ**	**6**
Kilic, 2023	**ΡΡ**		**ΡΡ**	**4**
Rynja, 2018	**ΡΡΡ**	**Ρ**	**ΡΡΡ**	**7**
Andersson, 2016	**ΡΡΡ**	**Ρ**	**ΡΡ**	**6**
Majstorovic, 2020	**ΡΡΡ**	**Ρ**	**ΡΡ**	**6**
Anttila, 2025	**ΡΡΡ**		**ΡΡ**	**5**
Örtqvist, 2017	**ΡΡΡ**	**Ρ**	**ΡΡΡ**	**7**
Akslund, 2009	**ΡΡΡΡ**	**Ρ**	**ΡΡΡ**	**8**
Kumar, 2016	**ΡΡΡΡ**	**Ρ**	**ΡΡΡ**	**8**
Nordenvall, 2020	**ΡΡΡΡ**	**Ρ**	**ΡΡΡ**	**8**

### Data synthesis and heterogeneity considerations

Clinical heterogeneity resulted from variations in hypospadias classification, age at repair, number of surgical procedures, and age at follow-up. Methodological heterogeneity was observed due to differing study designs, sample sizes, and follow-up periods. Outcome heterogeneity was particularly prominent, as sexual function and fertility were assessed using diverse instruments and non-standardized endpoints. Because of these differences, outcome measures could not be meaningfully combined or directly compared. Consequently, quantitative meta-analysis was not feasible, and the results of this review were synthesized using a qualitative descriptive approach. We also discuss how this variability limits direct comparison across studies and may influence the generalizability of the findings.

### Sexual function evaluation instrument

Across the included studies, various validated instruments were used to assess sexual function in adolescents and adults following hypospadias repair. Tack et al. used the International Index of Erectile Function (IIEF-5) to screen for erectile dysfunction in adolescents aged 16–21 years, and in a follow-up study applied the broader IIEF-15 to evaluate erectile and ejaculatory function in the same cohort [11, 12]. Kilic et al. assessed sexual function using the Arizona Sexual Experience Scale (ASEX) in adults with distal and proximal repairs [13]. Husmann et al. evaluated erectile function using the Sexual Health Inventory for Men (SHIM) in a large adult cohort with multiple prior repairs [14]. Rynja et al. used the IIEF-15, International Prostate Symptom Score (IPSS), and NRV genital perception questionnaire in men aged ≥ 18 years, and in a subsequent comparison of proximal vs. distal repairs and controls, again applied the IIEF-15 [15, 16]. Additional psychosexual assessments included the Penile Perception Score (PPS) in the study by Andersson et al. and the Global Sexual Functioning (GSF) questionnaire in the study by Majstorovic et al. [17, 18]. Overall, the heterogeneity of measurement tools—ranging from focused erectile function instruments to broader psychosexual questionnaires—highlights substantial variability in outcome assessment across studies. Anttila et al. evaluated postoperative sexual function in adolescents using a structured questionnaire that included the Erection Hardness Score (EHS) to assess erection rigidity, along with additional questions regarding penile straightness, ejaculation, and penile pain. Their instrument focused primarily on erection quality and basic sexual function milestones rather than comprehensive domains such as intercourse satisfaction or orgasmic function [19].

### Sexual dysfunction

Sexual function after hypospadias repair involves multiple domains, including erectile performance, ejaculatory function, orgasmic capacity, genital sensitivity, and overall sexual satisfaction. The evidence demonstrates substantial heterogeneity across these domains, largely influenced by the severity of hypospadias, number of prior surgeries, and specific surgical techniques.

Most studies report that erectile function in individuals with repaired hypospadias—particularly distal forms is generally comparable to that of controls. Tack et al. observed mild erectile dysfunction in approximately 10–12% of patients, with no significant association between meatal position and IIEF-5 scores [11, 12]. Similarly, Örtqvist et al. found high rates of erection satisfaction across distal, midshaft, and proximal groups, with occasional erectile difficulty reported in only 6–10% [20].

However, patients with proximal hypospadias or a history of multiple failed operations experience substantially poorer erectile outcomes. Kilic et al. reported symptomatic erectile dysfunction in up to 75% of proximal cases [13]. Husmann et al. documented moderate–severe ED (SHIM score 16) in 37% of patients with multiple prior repairs, identifying risk factors such as urethral plate division and ventral corporal grafting. These factors may predispose to early-onset ED through mechanisms including smooth-muscle dysfunction, venous leak phenomena, or graft-related corporal tissue alterations. Psychogenic contributors—such as anxiety and sexual inhibition related to repeated surgeries—are also highlighted [14].

### Ejaculatory function

Ejaculatory dysfunction is one of the most consistently reported sequelae, particularly in proximal hypospadias. Tack et al. noted ejaculatory problems—including weak ejaculation, split ejaculation, pain, and anejaculation—in approximately 12% of cases, with significantly higher combined sexual dysfunction (erectile and ejaculatory) in proximal versus distal repairs [11, 12]. Örtqvist et al. similarly reported increasing ejaculatory problems with severity: 1% in distal, 7.5% in midshaft, and 9% in proximal hypospadias.[20]. Rynja et al. observed weak ejaculation primarily among proximal repairs, although this did not correlate with orgasmic scores or other IIEF domains [15]. In the Anttila cohort, 95% of adolescents reported normal ejaculation, with no evidence of anejaculation or significant disturbances [19].

### Orgasmic function

Several studies identify reduced orgasmic function after hypospadias repair, particularly in proximal cases. Rynja et al. demonstrated significantly lower orgasmic frequency and reduced orgasmic sensation in the hypospadias group compared with controls, with more pronounced symptoms in proximal defects [16]. However, in patients treated with modern proximal techniques such as TPIT, Rynja et al. reported orgasmic outcomes similar to distal repairs and controls, despite persistently lower sexual desire or orgasmic intensity in some patients [15]. Majstorovic et al. found increased scores in domains related to sexual activity, arousal, and orgasmic abilities, suggesting that orgasmic function may remain preserved or even heightened in certain cohorts [18].

### Glanular sensitivity and penile sensation

Altered genital sensation is a recurrent finding. Örtqvist et al. reported significantly reduced glanular sensitivity among hypospadias patients, with a clear gradient of worsening sensation from distal to proximal forms. Despite this, nearly all patients remained capable of orgasm. Pain during intercourse—largely attributable to scarring, fissuring, or bleeding—was also described in the same cohort. Although fewer studies address this domain directly, the available evidence suggests that sensory disturbances are more common in proximal and repeatedly operated cases [20].

### Penile curvature, body image, and psychosexual outcomes

Residual penile curvature during erection remains common, particularly among proximal repairs. Tack et al. reported curvature in 81% of patients, contributing substantially to suboptimal sexual outcomes [11]. Dissatisfaction with penile length is also more prevalent in proximal hypospadias, a finding supported by Andresson et al., who noted limited penile growth during puberty in this group compared with distal and control groups [17]. Psychosexual concerns—such as anxiety about penile appearance, sexual inhibition, and reduced confidence—are closely linked to the number of operations and total duration of surgical treatment, as described by Husmann et al. [14] Anttila et al. reported highly favorable anatomical and psychosexual outcomes, with 95% of adolescents describing their penis as straight during erection and only 3% reporting any penile pain, none of which occurred during erection [19]. Participants also demonstrated high satisfaction with penile appearance, independent of hypospadias severity or prior curvature correction. These findings indicate that childhood repair generally yields reassuring long-term aesthetic and psychosexual outcomes across adolescence and adulthood.

Synthesizing findings across domains, distal hypospadias repairs generally yield sexual outcomes comparable to those of healthy controls, as demonstrated by several studies [13, 20]. In contrast, proximal hypospadias is consistently associated with higher rates of erectile dysfunction, ejaculatory abnormalities, sensory changes, curvature, and dissatisfaction with penile appearance. Tack et al. reported that combined adverse outcomes were more than twice as common in proximal than distal repairs [11]. Smaller studies, such as Diaw et al., demonstrate variable IIEF scores but do not alter the overall conclusion that proximal hypospadias carries a substantially higher risk of long-term sexual dysfunction [6].

### Fertility after hypospadias repair

Fertility outcomes after hypospadias differ substantially depending on the presence of associated disorders, hypospadias severity, and reproductive endocrine status. The evidence demonstrates that isolated hypospadias is largely compatible with normal reproductive potential, whereas proximal hypospadias and cases associated with cryptorchidism or testicular pathology show measurable impairment.

### Semen quality and testicular function

Akslund et al. conducted one of the largest comparative studies, evaluating 112 Danish men with a history of hypospadias and 318 age-matched controls. Semen parameters among men with isolated hypospadias (IH) were comparable to controls, suggesting preserved spermatogenesis in this group. In contrast, men with hypospadias accompanied by cryptorchidism and/or germ-cell neoplasia (HAGD) exhibited significantly reduced sperm concentration (median 52 vs. 32 million/mL, *p* = 0.02), total sperm count (173 vs. 101 million, *p* = 0.03), and normal sperm morphology (9% vs. 4%, *p* = 0.04). Semen volume and sperm motility did not differ significantly. These findings are consistent with impaired testicular function, further supported by the observation of smaller testicular volumes in the HAGD group (19.5 mL vs. 23.0 mL, *p* = 0.02). Hormonal profiles demonstrated elevated LH and FSH and lower testosterone levels in both IH and HAGD relative to controls, though the inhibin B/FSH ratio was particularly reduced in HAGD (*p* = 0.02), reflecting more pronounced Sertoli cell dysfunction [21].

Kumar et al. similarly reported compromised semen parameters among 73 men with hypospadias or a history of childhood repair. Proximal cases had significantly lower semen volume, sperm concentration, motility, and morphology compared with controls (*p* < 0.05). Comparisons across hypospadias subtypes revealed that semen parameters were largely similar, except for semen volume, which remained poorest in proximal defects. Endocrine evaluation showed normal hormonal ranges overall; however, FSH and LH were significantly elevated and testosterone lower than in controls (*p* < 0.001), consistent with subtle testicular function compromise. Testicular size did not differ significantly. Notably, semen and hormonal profiles were comparable between men repaired in childhood versus adulthood, although penile length was shorter in those operated in childhood (*p* = 0.01 flaccid, *p* = 0.007 erect). Nonprojectile ejaculation was common in proximal hypospadias (62.5%), and half of these cases involved childhood repairs [4].

### Paternity, fertility rates, and population-level outcomes

Self-reported fertility outcomes from Örtqvist et al. involving 167 men with hypospadias compared with both general and circumcised controls, showed no overall fertility difference between hypospadias and the general population. However, proximal hypospadias was associated with significantly lower fertility compared with both distal hypospadias (*p* = 0.001) and general controls (*p* = 0.002). Increasing severity of hypospadias was also associated with reduced likelihood of having a partner. Paternity rates were markedly reduced in proximal cases (19%) compared with midshaft (52%) and distal (47.5%) hypospadias (*p* = 0.005) [20].

Population-based data from Nordenvall et al. (21) further reinforce the severity-dependent gradient in reproductive outcomes. Using Swedish national registry data, the authors showed that distal hypospadias did not confer an increased risk of infertility (HR 1.17), whereas proximal hypospadias was associated with a markedly elevated risk (HR 3.59). The overall infertility diagnosis rate was 1.8% among men with hypospadias, rising to 3.1% in proximal cases and 2% in distal cases, compared with 1.6% in controls. Paternity was significantly lower in men with hypospadias (21.2%), especially among proximal cases (10.4%), whereas distal cases achieved 27.4%. When compared with their biological brothers, however, paternity rates and infertility diagnoses were similar (22.7% vs. 23.7% for paternity; 2% vs. 1.3% for infertility), suggesting shared familial or genetic factors contributing to observed outcomes. Use of assisted reproductive technologies (ART) was highest in proximal hypospadias (OR 6.95), indicating clinically significant reproductive impairment in this subgroup [22].

Taken together, the available evidence demonstrates that isolated hypospadias generally does not impair semen quality or fertility, whereas proximal hypospadias and hypospadias associated with cryptorchidism or testicular pathology show consistently worse semen parameters, higher gonadotropin levels, lower testosterone, and reduced paternity. The severity of hypospadias appears to be the strongest predictor of long-term reproductive outcomes, while some population-level findings suggest possible shared genetic or developmental contributors affecting both hypospadias and fertility.

## Discussion

One of the most crucial indicators of male sexual development is the proper growth of the penis [23] The reduced penile size in hypospadias patients may be the only cause of penile dissatisfaction, and it may be brought on by a number of circumstances including lower levels of androgens, the existence of undescended testicles, surgical damage, and an insufficient repair of penile curvature [20, 24]. Various studies have reported varying degrees of hypospadias’ impact on penile development [25]. Andresson et al. found that adolescents with proximal type hypospadias regarding psychosexual development and sexual function was comparable to control. Despite concerns about shorter penile length, proximal type group had engaged in sexual activity to same extent with distal group and control.^17^.

Hypospadias males who served as the control group in the current study reported good sexual desire, proving that the original penile deformity had no bearing on libido potency [4]. A study by Kanematsu et al. showed that in hypospadias patients, the sexual activity and marriage rate were not significantly different from those of the normal healthy group [26]. Majstorvic et al. found that the values for orgasmic ability (*p* = 0.002), arousal (*p* = 0.033), and sexual activity (*p* = 0.017) were all considerably higher in patients. Sexual activity and desire were found to be strongly correlated (*R* = 0.872), as were arousal and desire (*R* = 0.753), and orgasmic and erectile function (*R* = 0.769). Different psychosexual functioning areas in the patient group showed varying degrees of correlation with one another, leading to a varied manifestation of psychosexual dysfunctions [18].

Most study suggest that hypospadias degree is not correlated with erectile function. Rynja et al. found the mean IIEF-15 scores between study and control did not significantly differ except orgasmic function that significantly lower in proximal group compared to control [11, 12]. Another study by Majstorovic et al. found non-significant difference between control and patients in erectile ability domain [18]. Diaw et al. also found out of 3 patients, sexual function was good in one patient and average in the other two [6]. Similar result also stated by Attila et al. that erectile rigidity and penile straightness in adolescence and generally the same with control normal group, even in proximal cases [19]. Notably by Kilic et al. state that the distal hypospadias group has sexual, erectile, and ejaculatory activities that are comparable to those of healthy normal people, however, there is a greater risk of ejaculation issues and around 75% of individuals in the proximal hypospadias group had symptomatic erectile dysfunction [13]. Tack et al. suggest that proximal type hypospadias prone to have suboptimal sexual function due to penile curvature combined with erection or ejaculatory problem along with urinary disturbance which lead to reintervention and poor psychosexual development [11]. The impact and prevalence of psychogenic ED within general hypospadias especially with multiple attempt failure surgery should also be considered [14].

Rynja et al. pointed out that patients with proximal type hypospadias suffer with worse ejaculatory function and orgasmic sensation than control [15, 16]. Ejaculatory compromised might be explained by the neourethra or persistent utricle. Anejaculation also reported by 11% of proximal hypospadias patient in Anderssons et al. study [17]. Örtqvist et al. also found anejaculation in each group type of hypospadias groups [20].

Excellent libido and good quality of penile erection are more impotent part of sexual life. As the quality of erection was comparable between distal type and control but may significantly lower in proximal group, overall level of satisfaction of both patient and partner sexual activity did not differ significantly from control in Kumar et al. study [4]. As a result, among those who underwent hypospadias surgery, those who had distal surgery experienced sexual pleasure at a rate comparable to that of adult healthy people. This data suggests that the general sexual function was not affected by hypospadias, or the surgery associated with it. Another factor associated with sexual function is reoperation. Kanematsu et al. found that patients who underwent a second operation for an obstructive complication had poorer International Prostate Symptom Score scores and more issues with ejaculation. Stojanofic et al. also stated that one-stage repair had a greater success rate. Four occurrences of late-onset penile curvature, one case of glans dehiscence, and partial skin necrosis were among the complications. 24% of individuals had erectile dysfunction identified [26].

Theoretically, hypospadias can be associated with impaired testicular function that leads to subfertility and infertility. In the development of hypospadias, the actual fertility potential of patient with hypospadias has not been clearly defined. Semen sample and paternity rates (become biologically father) can be the key element, but the usage of ART, reproductive hormones, and diagnosis of infertility should also be considered.

Study conducted by Kumar et al. found that patient with proximal hypospadias had poorer semen quality compared to control especially lower sperm density, percentage sperm motility, and normal morphology. Distal hypospadias had comparable semen quality parameter with control [4]. This result support Asklund et al. study, that male with proximal type had comparable semen parameter to control but significantly lower in proximal type group [21].

Fertility in male with hypospadias may be affected due to various factor such as anatomical, surgical, and associated disorder. Therefore, fertility issue should be considered as multifactorial [27]. Implied that ejaculatory problem in male after hypospadias rages between 4 and 63%, infertility can occur due to inability to project semen into cervix of partner. A sufficient amount of semen must be deposited in the female vaginal canal in order for the eggs to fertilize, and this relies on the way the male partner ejaculates [28]. More frequently than the distal type group of hypospadias patients (4.10%), proximal hypospadias patients had ejaculation issues, such as weak or dribbling ejaculation or the need to milk out ejaculate after sexual activity. These issues might be brought on by skin folds, related urethral diverticula, aberrant prostate, and seminal vesicle growth, or a greater incidence of postoperative urethral stricture [4].

Proximal type of hypospadias was said to have impaired masculinization with endocrine diminishment of hypothalamic-pituitary-gonadal axis. Glandular and penile shaft positioned meatus are considered to have less impact on gonadal function. The presence of descendent testes also impair the fertility potential of male especially in hypospadias cases [21]. Studies suggest that FSH might serve as possible marker of spermatogenic function [29]. In Kumar et al. study, the mean value of both serum FSH and LH were significantly higher with lower testosterone level in hypospadias patient than control. Hormonal disturbances were seen more in proximal type group which lead to poorer semen quality [4]. However, methodological bias may exist due to small sample size.

Study by Asklund et al. support the fact that a decreased Leydig cell and Sertoli cell function in most hypospadias that associated micro-penis, cryptorchidism, and ambiguous genitalia. In fact isolated hypospadias may not be caused by testicular dysgenesis but other factor that interfering development stage during penile development and cause no hormone disturbance during adult life [21]. Study by Nordenvall et al. result support previous study that as a whole group, men with hypospadias were less likely to conceive biological offspring especially proximal type compared to distal and control. Assisted reproductive technology (ART) indicates subfertility pattern. Although this technology made fatherhood more common for men with hypospadias than it was for those who also had cryptorchidism, risk of using ART was elevated regardless of concomitant cryptorchidism suggesting that anatomical factors linked to hypospadias increase the need of ART rather than impaired semen quality [22]. On contrary, study conducted by Örtqvist et al. showed that hypospadias male have good sexual outcome and satisfaction with sexual life with comparable fertility. Paternity level of hypospadias patients was comparable to control with usage of ART was rare. In this study, only patient with proximal hypospadias presented with lower paternity level [20].

One noteworthy limitation of this literature evaluation was that we cannot fully compare the outcomes of each type of hypospadias as not all literature we found have data for each type of hypospadias. Limited study that compares sexual function to control also become one of the limitations that we cannot perform deeper analysis and comparison. Significant methodological heterogeneity was also observed across studies, including differing hypospadias classification systems, variable follow-up periods, and use of multiple sexual function instruments. These differences limit direct comparison between studies and may contribute to variability in reported outcomes. The lack of standardization also restricts the ability to perform a meta-analysis. Variety of sexual function instruments is also one of confounding factors that limit the analysis of this review. Furthermore, many included studies were limited by small sample sizes, cross-sectional designs, heterogeneous questionnaires, low response rates, and inconsistent definitions of sexual dysfunction. These limitations restrict direct quantitative comparison and may introduce imprecision or reporting bias. As a result, the findings of this review should be interpreted as qualitative synthesis rather than a quantitative estimate of effect, and the conclusions should be considered with appropriate caution. Further study with variety of social economic backgrounds that compare different types of hypospadias and healthy people should be conducted to validate this review.

## Conclusion

Over the last three decades, the prognosis of hypospadias has significantly improved, but there are persistent psychological concerns. In our investigation, the distal hypospadias group has more favorable sexual function, erectile function, and ejaculatory activities outcome than proximal hypospadias; however, meaningful rates of dysfunction remain even in distal cases. The findings should not be interpreted as equivalent to the normal population but rather as relatively better outcomes within the hypospadias spectrum. The proximal variety, however, exhibited suboptimal sexual function due to penile curvature which disturbs erection, low semen quality and ejaculation function. To support the conclusions and guide clinical management effectively, further multicentric research is required.

## Supplementary Information

Supplementary Material 1.

## Data Availability

No datasets were generated or analyzed during the current study.

## Abbreviations

ART: Assisted reproduction technology
ASEX: Arizona Sexual Experience scale
ED: Erectile dysfunction
FSH: Follicle stimulating Hormone
GSF: Global sexual functioning
HAGD: Hypospadias, cryptorchidism, and/or germ cell neoplasia
IH: Isolated Hypospadias
IIEF: International Index of Erectile Function
LH: Luteinizing hormone
NOS: Newcastle-Ottawa Scale
PPS: Penile perception score
PRISMA: Preferred Reporting items for Systematic Review and Meta-Analysis
TPIT: Transverse preputial island tube
